# Therapeutic Potential of N-acetylcysteine and Glycine in Reducing Pulmonary Injury in Diabetic Rats

**DOI:** 10.7759/cureus.72902

**Published:** 2024-11-02

**Authors:** Malik Ejubović, Dina Kapic, Samra Custovic, Edina Lazović Salčin, Orhan Lepara, Avdo Kurtović, Rijad Jahić, Aida Kulo Cesic, Belma Paralija, Nermina Ziga Smajic, Amira Jagodić Ejubović, Snijezana Hasanbegovic, Muhamed Katica, Aida Besic, Enra Djesevic, Almir Fajkić

**Affiliations:** 1 Internal Medicine, Cantonal Hospital Zenica, Zenica, BIH; 2 Histology and Embryology, University of Sarajevo, Sarajevo, BIH; 3 Pathology, University of Sarajevo, Sarajevo, BIH; 4 Human Physiology, University of Sarajevo, Sarajevo, BIH; 5 Orthopedics and Traumatology, Tuzla University Clinical Center, Tuzla, BIH; 6 Internal Medicine and Cardiology, Sarajevo University Clinical Center, Sarajevo, BIH; 7 Pharmacology, University of Sarajevo, Sarajevo, BIH; 8 Pulmonology, Sarajevo University Clinical Center, Sarajevo, BIH; 9 Pharmacy, University of Sarajevo, Sarajevo, BIH; 10 Pediatrics, Sarajevo University Clinical Center, Sarajevo, BIH; 11 Clinical Disease, University of Sarajevo, Sarajevo, BIH; 12 Veterinary Medicine, University of Sarajevo, Sarajevo, BIH; 13 Endocrinology, Sarajevo University Clinical Center, Sarajevo, BIH; 14 Pathophysiology and Internal Medicine, University of Sarajevo, Sarajevo, BIH

**Keywords:** diabetes mellitus, glycine, inflammation, lung injury, n-acetylcysteine, oxidative stress

## Abstract

Introduction: Diabetes mellitus is associated with systemic complications, including the development of pulmonary injury, characterized mainly by excessive accumulation of extracellular matrix components and inflammatory cell infiltration in lung tissue. This process is driven by oxidative stress and chronic inflammation, both caused and exacerbated by hyperglycemia. N-acetylcysteine (NAC) and glycine, known for their antioxidant and anti-inflammatory effects, offer potential therapeutic benefits in mitigating diabetes-induced lung injury.

Objective: The study aimed to investigate the effects of supplementation by either NAC or glycine or their combination on reducing lung injury in rats with type 1 diabetes

Materials and methods: The study used 30 adult Wistar albino rats (10 weeks old, weighing between 180 g and 380 g). Six of them were used as controls, while 24 adult rats (10 weeks old, 180-380 g) with type 1 diabetes, induced through a single intraperitoneal injection of streptozotocin (STZ) at a dose of 55 mg/kg, were randomly assigned to four experimental groups: control (CTL), diabetic (Db), NAC treatment (diabetic+NAC), glycine treatment (diabetic+glycine), and combined NAC and glycine treatment (diabetic+NAC+glycine). NAC (100 mg/kg) and glycine (250 mg/kg) were administered orally for 12 weeks. At the end of the study, lung tissues were collected for histopathological examination. Qualitative, semi-quantitative, and stereological histological analysis was used to analyze structural changes in the lung tissue. Semi-quantitative scoring was carried out to evaluate the extent of inflammation, while stereological analysis was performed to determine the volume density of alveolar spaces and septal connective tissue. The semi-quantitative scoring included scores ranging from 0 (absent), 1 (minimal), 2 (mild), 3 (moderate), to 4 (severe).

Results: Qualitative histological analysis revealed pronounced inflammation and fibrosis in the lungs of untreated diabetic rats, characterized by thickened alveolar septa and immune cell infiltration. Both treatments with NAC and glycine individually reduced inflammation and fibrosis compared to untreated diabetic rats. The greatest improvement was observed in the NAC+glycine group, where the alveolar structure appeared almost normal, with minimal inflammation. Semiquantitative analysis showed statistically significant differences in peribronchial and peribrochiolar infiltrates between the diabetic group (2.16±0.47) and the control group (0.33±0.21, p=0.026). The combination of NAC and glycine significantly reduced peribronchial and peribronchiolar infiltrates (0.33±0.33, p=0.026) compared to the diabetic group. Similarly, septal inflammatory infiltrates were significantly lower in the NAC+glycine group (1±0.36) compared to diabetic rats (3.33±0.33, p=0.004). Total airway inflammatory infiltration was also significantly reduced in the NAC+glycine group (1.33±0.33, p=0.002) compared to the diabetic group (5.5±0.5).

Conclusion: As the combination of NAC and glycine demonstrated protective effects against lung inflammation and fibrosis in diabetic rats, a synergistic effect of NAC and glycine in mitigating pulmonary complications associated with type 1 diabetes may be suggested. These findings warrant further exploration of the combination for managing diabetic lung disease and potentially other fibrotic conditions.

## Introduction

Diabetes mellitus, a common metabolic disease marked by persistent high blood sugar levels, is now mainly acknowledged for its wide range of serious consequences that impact several organs and systems. Diabetes can influence multiple organ systems, including the cardiovascular, renal, nervous, and respiratory systems, leading to a range of complications such as cardiovascular disease, nephropathy, neuropathy, and pulmonary issues. The development of pulmonary fibrosis, a progressive disease characterized by excessive accumulation of extracellular matrix components in the lung interstitium, is a notable but sometimes disregarded consequence. Over time, this process results in a progressive decline in lung function and overall quality of life. Intricate pathways, including oxidative stress, chronic inflammation, and dysregulated immunological responses, are involved in the multidimensional connection between diabetes and pulmonary fibrosis [1].

Understanding and addressing these processes is crucial in the fight against diabetes and pulmonary fibrosis. The present difficulties in addressing diabetes-induced lung injury arise from the intricate pathophysiology connecting diabetes to pulmonary damage, encompassing interactions among hyperglycemia, inflammation, and oxidative stress. The paucity of research in this domain, along with patient heterogeneity, concomitant conditions, and the adverse effects of diabetic drugs, hampers the formulation of effective treatment methods and demands greater exploration of targeted therapeutic alternatives.

The role of oxidative stress in the development of diabetes complications cannot be overlooked. Chronic hyperglycemia causes an excessive generation of reactive oxygen species (ROS), which can overpower the body's antioxidant mechanisms and damage cells. Oxidative stress in the lungs explicitly triggers the activation of fibroblasts and myofibroblasts, essential cells in the fibrotic process. Moreover, the oxidative stress associated with diabetes is intensified by systemic inflammation, hence worsening tissue damage and fibrosis [2,3]. Furthermore, chronic inflammation, a key player in the progression of lung fibrosis, is significantly elevated in individuals with diabetes. This is evidenced by the increased concentrations of pro-inflammatory cytokines such as interleukin-6 (IL-6). These cytokines sustain inflammatory reactions, promote the growth of fibroblasts, and contribute to the accumulation of collagen in the lungs. This chronic inflammatory environment establishes a harmful cycle that accelerates the advancement of fibrosis, underscoring the severity of the issue [4].

On the other hand, N-acetylcysteine (NAC) and glycine with their potent antioxidative and anti-inflammatory properties are emerging as promising therapeutic agents in patients with diabetes-related complications [5]. NAC, a well-recognized precursor to glutathione, one of the body's most potent antioxidants, can reduce oxidative damage and regulate inflammatory reactions that may diminish fibrotic alterations in lung tissue. Similarly, glycine, an amino acid with remarkable anti-inflammatory properties, can modulate immune responses and suppress the synthesis of inflammatory cytokines [4-6]. Also, their combination shows considerable therapeutic potential in treating oxidative stress and inflammation, but also several other pathophysiological processes including glutathione deficiency, mitochondrial dysfunction, physical function, and aging [7]. However, the data on the potential of this combination in reducing pulmonary fibrosis in patients with diabetes mellitus are scarce.

The study aimed to investigate the effects of supplementation with either NAC, glycine, or their combination on reducing lung fibrosis in rats with type 1 diabetes. Given the known associations between diabetes and the development of pulmonary complications, including lung fibrosis, this research seeks to explore whether these supplements can mitigate such adverse effects. By assessing the impact of NAC and glycine, both of which have been linked to antioxidant and anti-inflammatory properties, we aimed to determine their efficacy individually and in synergy, potentially providing insights into novel therapeutic strategies for managing lung fibrosis in diabetic patients.

## Materials and methods

Thirty adult Wistar albino rats (10 weeks old, weighing between 180 g and 380 g) were included in the study. Six of them were used as controls (CTL, fed the basal diet and supplemented with water for injection), while 24 rats with previously induced type 1 diabetes were randomly assigned to four experimental groups of six animals: diabetic (Db, fed the basal diet and supplemented with water for injection), NAC treatment (diabetic+NAC, fed the basal diet supplemented with 100 mg/kg NAC), glycine treatment (diabetic+glycine, fed the basal diet supplemented with 250 mg/kg glycine), and combined NAC and glycine treatment (diabetic+NAC+glycine, fed the basal diet supplemented with 100 mg/kg NAC and 250 mg/kg glyicine).

The dose of NAC and glycine were selected based on the previous experiments [8-10]. The water for injection and solutions of NAC and glycine in water for injection were administered orally (via gavage) to rats once daily for 12 weeks after type 1 diabetes was confirmed.

Diabetes induction

To induce type 1 diabetes, streptozotocin (STZ) at a dose of 55 mg/kg was dissolved in citrate buffer (pH 4.5) and administered intraperitoneally (ip). STZ is commonly used to induce diabetes in rat models because it selectively targets and destroys the insulin-producing beta cells in the pancreas [8]. Blood glucose levels were measured 72 hours after the ip injection using a commercial glucometer. Rats with fasting blood glucose levels between 220 mg/dL and 250 mg/dL (12.2 mmol/L and 13.9 mmol/L) were considered to be diabetic.

Experimental design

Thirty rats with type 1 diabetes were randomly divided into five groups of six animals: control (CTL) group (fed the basal diet and supplemented with water for injection), untreated diabetic (Db) group (fed the basal diet and supplemented with water for injection), NAC group (fed the basal diet supplemented with 100 mg/kg NAC), glycine group (fed the basal diet supplemented with 250 mg/kg glycine), and NAC+glycine group (fed the basal diet supplemented with 100 mg/kg NAC and 250 mg/kg glyicine). The dose of NAC and glycine were selected based on previous experiments [8-11]. The water for injection and solutions of NAC and glycine in water for injection were administered orally (via gavage) to rats once daily for 12 weeks after type 1 diabetes was confirmed.

Lung tissue collection

At the end of the 12 weeks, the rats were deeply anesthetized using intramuscular injections of ketamine hydrochloride (80 mg/kg) and xylazine (10 mg/kg), ensuring at least 30 minutes of anesthesia. Euthanasia was performed using standard protocols, and the lungs were immediately harvested for further analysis.

Histopathological analysis

Lung tissues were fixed in 10% formalin for 24 hours, followed by dehydration, clearing, and embedding in paraffin. Tissue sections of 5 µm thickness were prepared and stained with Hematoxylin and Eosin (H&E) for general morphological evaluation and Masson’s trichrome stain to assess fibrosis. These methods are standard practices commonly employed in this type of research. The stained sections were evaluated by scientists blinded to the experimental groups. Qualitative, semi-quantitative, and quantitative stereological histological examinations were carried out. Peribronchial and peribronchiolar infiltrate as well as septal inflammatory infiltrate were evaluated using a semi-quantitative grading system, with scores ranging from 0 (absent), 1 (minimal), 2 (mild), 3 (moderate), to 4 (marked) [12]. The total airway inflammatory infiltrate score was calculated as the sum of the previously mentioned infiltration scores. Volume density of the alveolar spaces and the septal connective tissue were determined by stereological analysis [13].

Statistical analysis

Statistical analysis was performed by two programs, MS Excel (Microsoft Office Excel 2010) and SPSS (Statistical Package for 27 Social Sciences) version 22.0 (IBM Corp., Armonk, NY). The Shapiro-Wilk test was used to assess the normality of variable distribution. The mean value (X) and standard error of the mean (SEM) were reported for the data. For continuous independent variables with normal distribution, the significance of differences was tested using the Student t-test, while for variables without normal distribution, the Mann-Whitney test was used. Differences among multiple groups of variables were assessed using analysis of variance (ANOVA) for normally distributed variables and the Kruskal-Wallis test for variables without normal distribution. A p<0.05 was considered statistically significant.

## Results

Qualitative histological analysis

**Figure 1:**
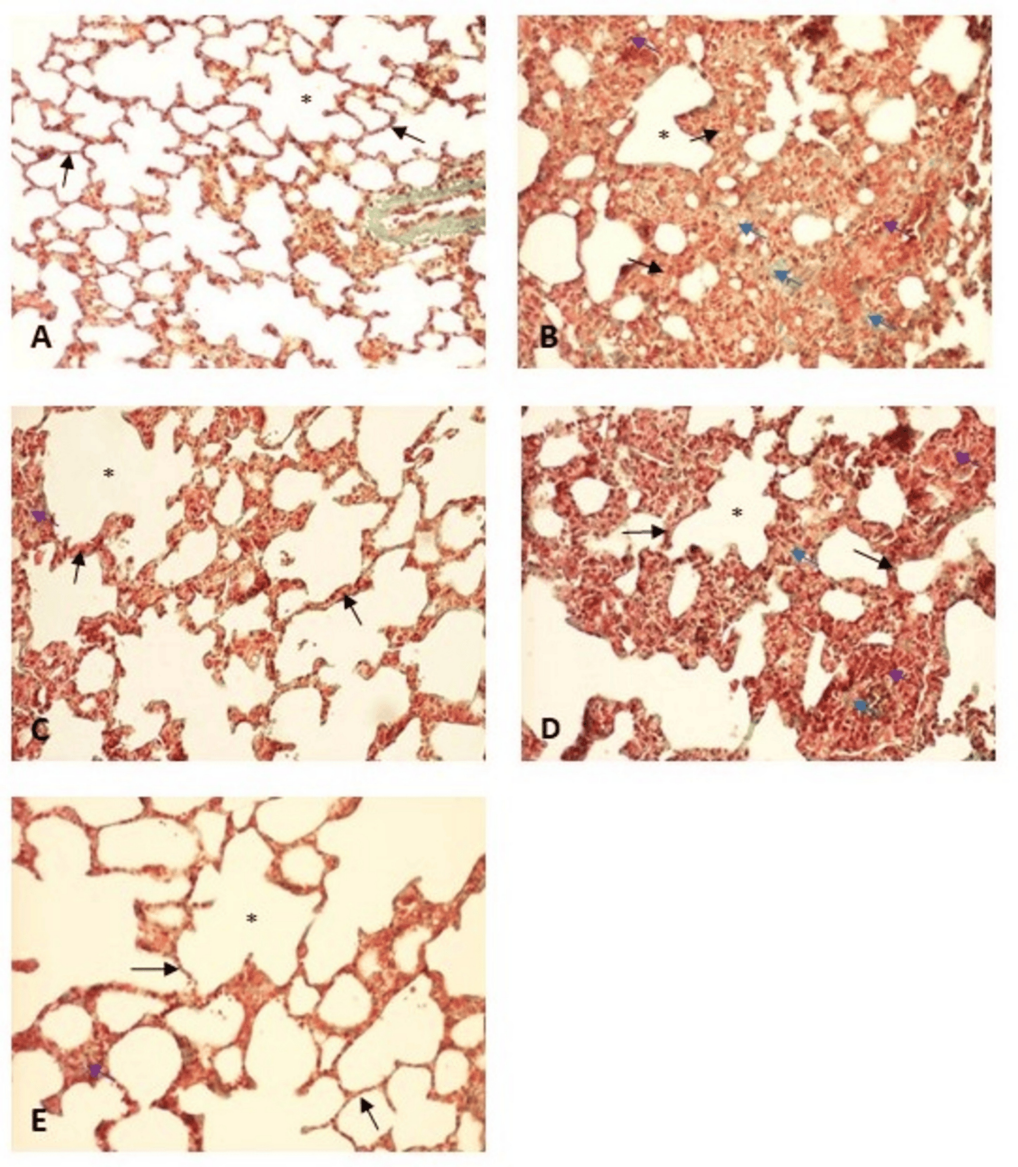
Representative photomicrographs of the lungs, Masson trichrome staining, 100x A: control group, B: group with diabetes mellitus, C: group with diabetes mellitus treated with NAC, D: group with diabetes mellitus treated with glycine, and E: group with diabetes mellitus treated with NAC and glycine. *, alveolar space; black arrow, alveolar septum; blue arrow, fibrosis; purple arrow, inflammatory cell infiltration; NAC, N-acetylcysteine

Qualitative histological analysis showed that the lungs in the control group exhibited a normal histological structure, with well-preserved alveoli, thin and uniform alveolar septa, and spacious alveolar spaces. There was no sign of thickening, fibrosis, or infiltration in the septal walls. In contrast, the diabetic rats displayed significant structural changes. The alveoli were irregularly shaped and markedly distorted, with a notable reduction in the alveolar air spaces. The alveolar septa were thickened and fibrotic and heavily infiltrated with immune cells, predominantly lymphocytes. In the lungs of diabetic rats treated with NAC for 12 weeks, the alveolar spaces were slightly less reduced than in untreated diabetic rats, although the septal walls remained moderately thickened. Lymphocyte infiltration persisted but was less pronounced, indicating a mild anti-inflammatory effect of NAC. Diabetic rats treated with glycine for 12 weeks exhibited less severe histological changes than untreated diabetic rats. The alveoli were less distorted, and the septal walls, while still infiltrated with mononuclear cells, showed decreased thickness. The septal connective tissue was more pronounced, indicating partial fibrosis, but the overall lung structure was better preserved compared to the untreated group. The lungs of diabetic rats treated with both NAC and glycine for 12 weeks demonstrated a near-normal histological appearance. The alveoli had a regular structure, with only minimal distortion, and the alveolar septa were relatively thin and only slightly infiltrated by immune cells. Inflammation was minimal, and the connective tissue was less pronounced than in other treated groups, suggesting that the combination of NAC and glycine had a synergistic protective effect on lung architecture (Figure 1A-1E). 

The results of semiquantitative and quantitative stereological analysis of the lungs in rats across different experimental groups are presented in Table 1. The presented results represent values for peribronchial and peribronchiolar inflammatory infiltrate, septal inflammatory infiltrate, airway inflammatory infiltrate, volume density of alveolar spaces, and volume density of septal connective tissue.

**Table 1:** The results of the semiquantitative and the quantitative stereological analysis in rats across different examined groups Data are presented as mean±SEM. CTL, control group; Db, group with diabetes mellitus; NAC, group with diabetes mellitus treated with NAC; glycine, group with diabetes mellitus treated with glycine; NAC+glycine: group with diabetes mellitus treated with NAC+glycine; NAC, N-acetylcysteine

Variable	CTL	Db	NAC	Glycine	NAC+glycine
Semiquantitative score					
Peribronchial and peribonchiolar infiltrate	0.333±0.21	2.16±0.47	2.16±0.54	1.83±0.61	0.33±0.33
Septal infiltrate	0.67±0.42	3.33±0.33	2.83±0.31	1.33±0.49	1±0.36
Total airway infiltrate	1±0.36	5.5±0.5	5±0.73	3.17±0.79	1.33±0.33
Stereological analysis					
Volume density of alveolar spaces	0.56±0.05	0.42±0.037	0.45±0.39	0.57±0.06	0.54±0.043
Volume density of the septal connective tissue	0.06±0.011	0.09±0.032	0.072±0.013	0.08±0.028	0.049±0.012

**Figure 2:**
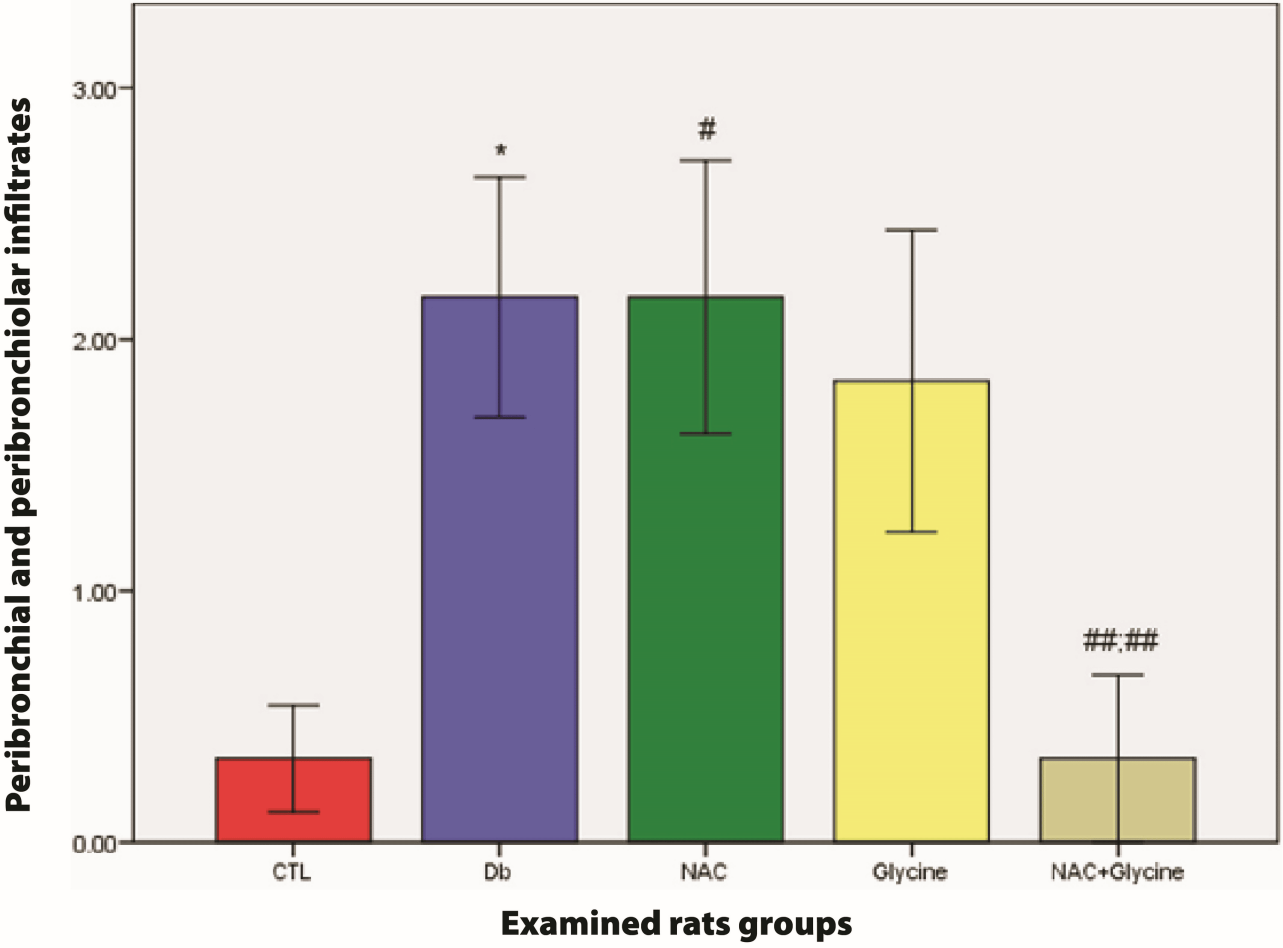
The mean values of peribronchial and peribronchiolar infiltrates of rats in examined groups Data are presented as mean±SEM. CTL, control group; Db, group with diabetes mellitus; NAC, group with diabetes mellitus treated with NAC; glycine, group with diabetes mellitus treated with glycine; NAC+glycine, group with diabetes mellitus treated with NAC+glycine; *, p<0.05 compared to control group; ^#^, p<0.05 compared to control group; ^##;##^, p<0.05 compared to NAC and Db group; NAC, N-acetylcysteine

Our analysis revealed a statistically higher value of peribronchial and peribronchiolar infiltrate in the Db (p=0.026) and NAC (p=0.027) groups compared to the control group. Similarly, there was a statistically lower value of peribronchial and peribronchiolar infiltrate between the NAC+glycine group, Db (p=0.026), and NAC (p=0.004) separately. However, there were no statistical differences between the other examined groups (glycine vs. CTL (p=0.132); NAC+glycine vs. CTL (p=0.818); NAC vs. Db (p=0.818); glycine vs. Db (p=0.818); NAC vs. glycine (p=0.699); NAC+glycine vs. glycine (p=0.093)) (Figure 2).

**Figure 3:**
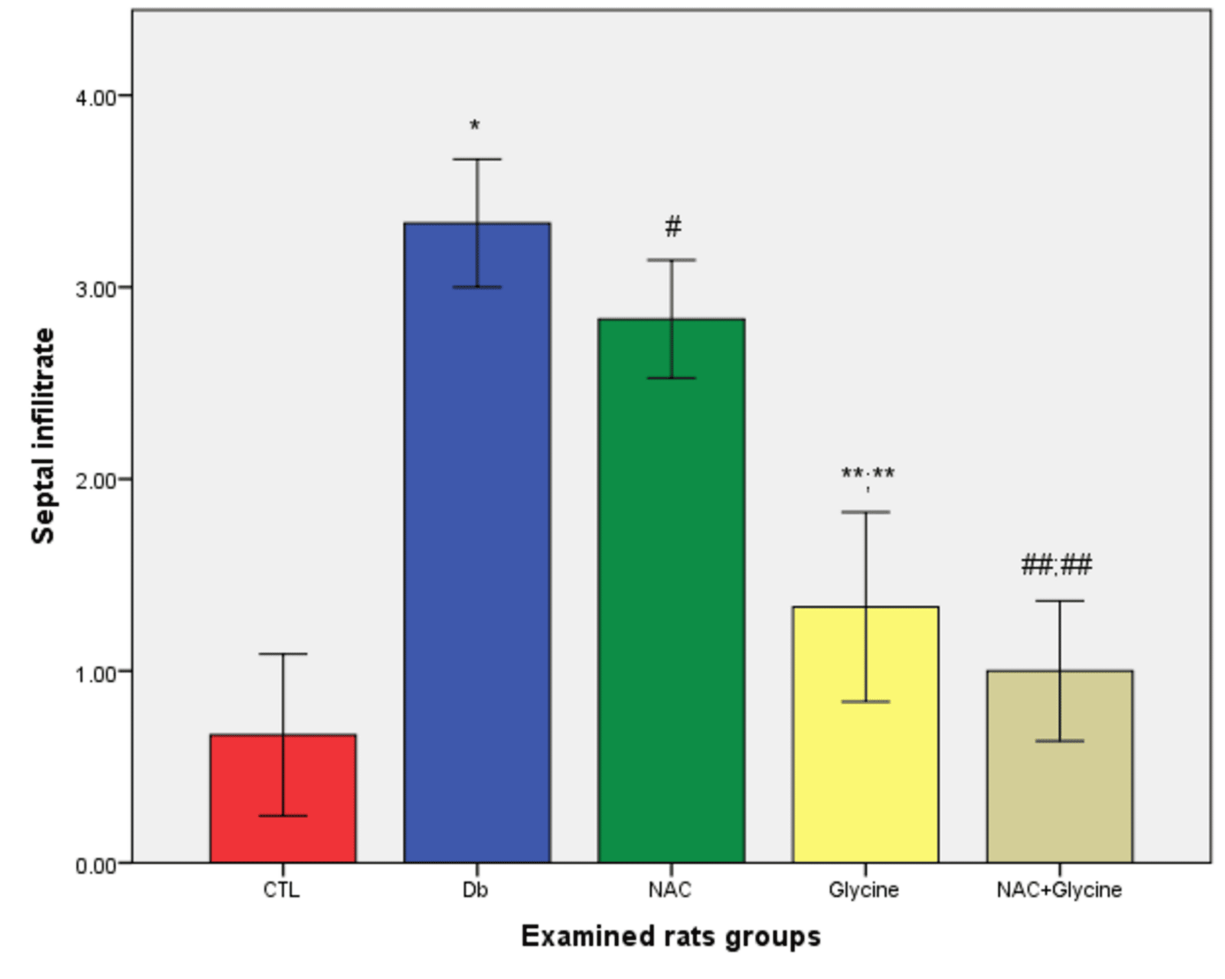
The mean values of septal infiltrates in examined groups Data are presented as mean±SEM. CTL, control group; Db, group with diabetes mellitus; NAC, group with diabetes mellitus treated with NAC; glycine, group with diabetes mellitus treated with glycine; NAC+glycine, group with diabetes mellitus treated with NAC+glycine; *, p<0.05 compared to control group; ^#^, p<0.05 compared to control group; **;**, p<0.05 compared to Db and NAC group; ^##;##^, p<0.05 compared to Db and NAC group; NAC, N-acetylcysteine

We found a statistically higher value of septal infiltrate in Db (p=0.004) and NAC groups (p=0.007) compared to the control group of rats; also, the level of the septal infiltrate was significantly lower in the glycine group compared to Db (p=0.015) and NAC (p=0.0.41) group separately. A statistically lower level of septal infiltrate was shown in the NAC+glycine group compared to Db (p=0.004) and NAC (p=0.026). Between other examined groups, there were no statistical differences (glycine vs. CTL (p=0.394); NAC+glycine vs. CTL (p=0.589), NAC vs. Db (p=0.310); NAC+glycine vs. glycine (p=0.699)) (Figure 3).

**Figure 4:**
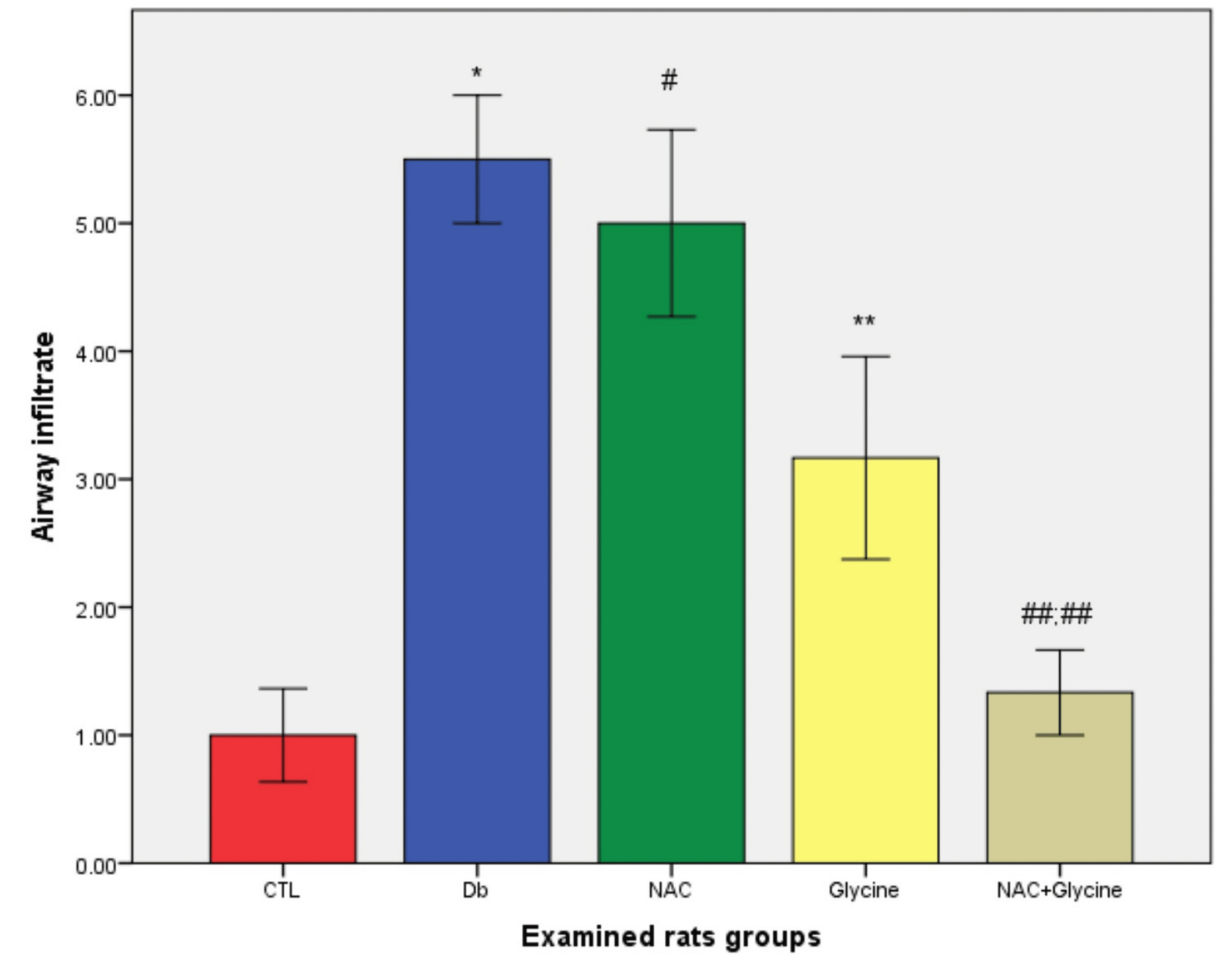
The mean values of airway infiltrates in examined groups Data are presented as mean±SEM. CTL, control group; Db, group with diabetes mellitus; NAC, group with diabetes mellitus treated with NAC; glycine, group with diabetes mellitus treated with glycine; NAC+glycine, group with diabetes mellitus treated with NAC+glycine; *, p<0.05 compared to control group; ^#^, p<0.05 compared to control group; **, p<0.05 compared to Db group; ^##;##^, p<0.05 compared to Db and NAC group; NAC, N-acetylcysteine

There was a statistically higher value of total airway infiltrate in Db (p=0.002) and NAC groups (p=0.004) compared to the control group; also, there was a statistically lower level of total airway infiltrate in the glycine group compared to the Db group (p=0.041; p<0.05). A statistically lower level of total airway infiltrate was shown in the NAC+glycine group compared to Db (p=0.002) and NAC (p=0.004) separately. Between other examined groups, there were no statistical differences (glycine vs. CTL (p=0.065); NAC+glycine vs. CTL (p=0.589); NAC vs. Db (p=0.818); glycine vs. NAC (p=0.132); NAC+glycine vs. glycine (p=0.093)) (Figure 4).

**Figure 5:**
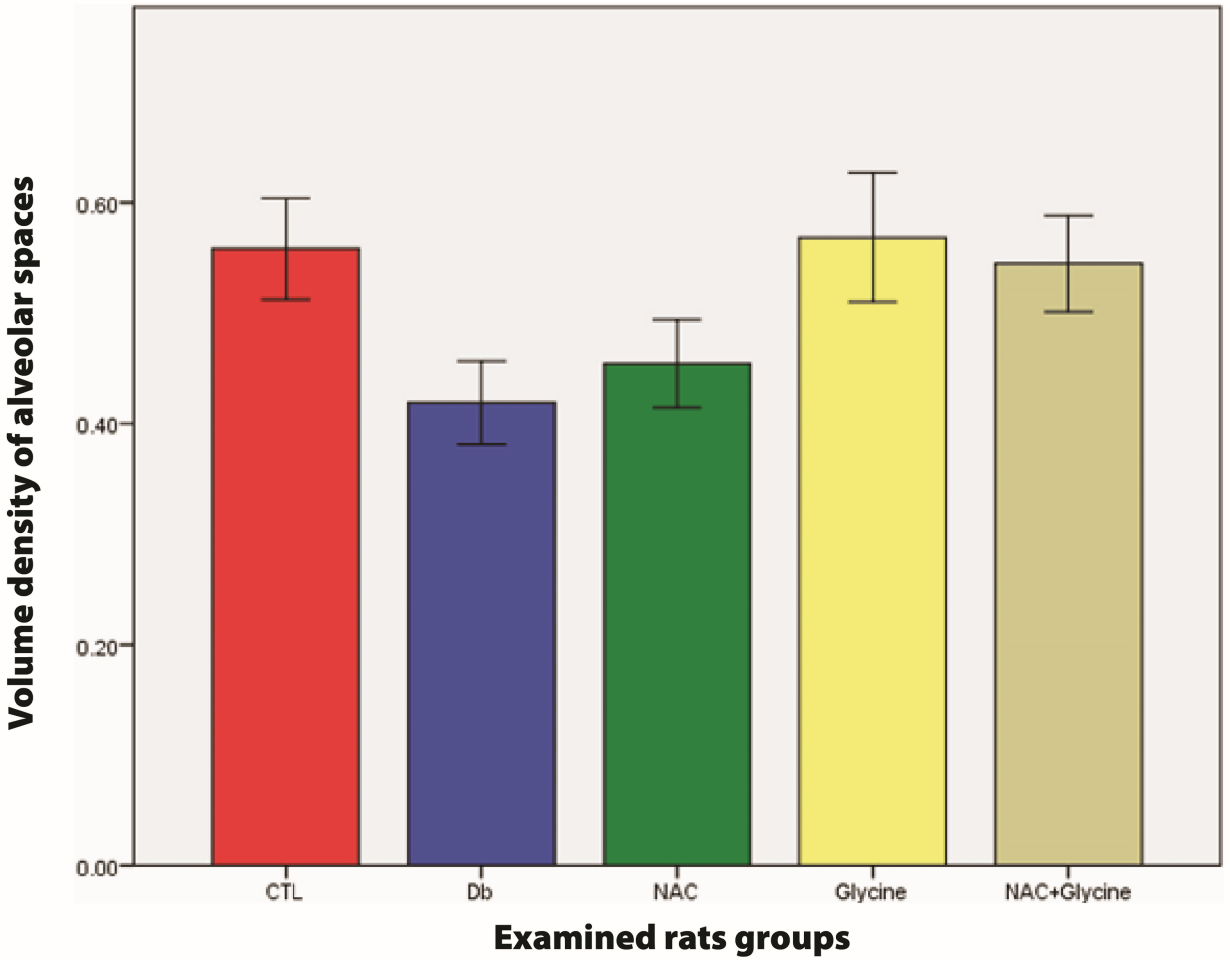
The mean values of volume density of alveolar spaces in examined groups Data are presented as mean±SEM. CTL, control group; Db, group with diabetes mellitus; NAC, group with diabetes mellitus treated with NAC; glycine, group with diabetes mellitus treated with glycine; NAC+glycine, group with diabetes mellitus treated with NAC+glycine; NAC, N-acetylcysteine

There was no statistical difference in volume density of alveolar spaces between examined groups (Db vs. CTL (p=0.065); NAC vs. CTL (p=0.180); glycine vs. CTL (p=0.456); NAC+glycine vs. CTL (p=0.654); NAC vs. Db (p=0.589); glycine vs. Db (p=0.093); NAC+glycine vs. Db (p=0.065); glycine vs. NAC (p=0.180); NAC+glycine vs. NAC (p=0.310); NAC+glycine vs. glycine (p=0.818)) (Figure 5).

**Figure 6:**
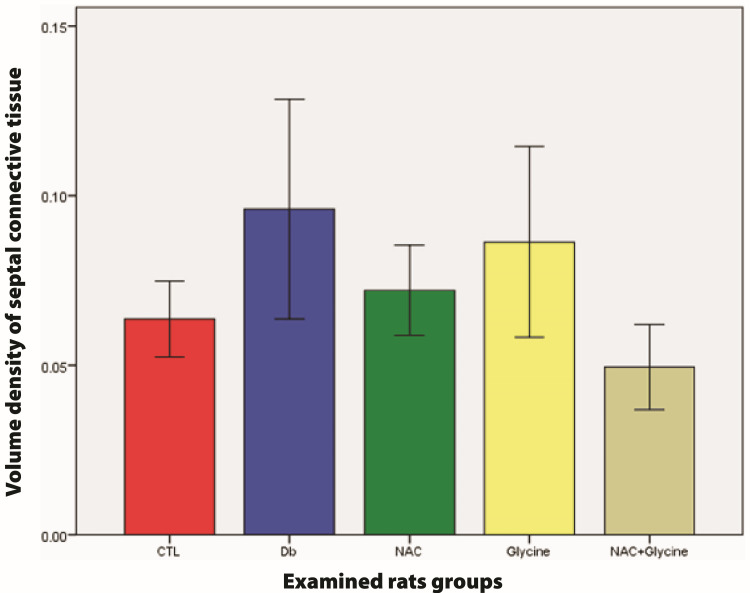
The mean values of volume density of septal connective tissue in examined groups Data are presented as mean±SEM. CTL, control group; Db, group with diabetes mellitus, NAC, group with diabetes mellitus treated with NAC; glycine, group with diabetes mellitus treated with glycine; NAC+glycine, group with diabetes mellitus treated with NAC+glycine; NAC, N-acetylcysteine

There was no statistical difference in the volume density of septal connective tissue between examined groups (Db vs. CTL (p=0.818); NAC vs. CTL (p=0.699); glycine vs. CTL (p=0.753); NAC+glycine vs. CTL (p=0.394); NAC vs. Db (p=0.818); glycine vs. Db (p=0.818); NAC+glycine vs. Db (p=0.589); glycine vs. NAC (p=0.818); NAC+glycine vs. NAC (p=0.240); NAC+glycine vs. glycine (p=0.485)) (Figure 6).

## Discussion

Compared to the single use of either NAC or glycine, both qualitative and semi-quantitative histopathological analyses confirmed their combination to be more effective in reducing pulmonary fibrosis in a diabetic rat model. Compared to no treatment, treatment with NAC showed only a mild anti-inflammatory effect, while treatment with glycine was less effective in reducing fibrosis and inflammation. In contrast, the combination of NAC and glycine resulted in an almost normal alveolar structure, with minimal inflammation observed. Also, the semi-quantitative analysis showed a significant reducement in peribronchial infiltrates in the NAC+glycine group (0.33±0.33) compared to the untreated diabetic group (2.16±0.47, p=0.004), as well as a significant reducement in septal infiltrates (1±0.36) and airway infiltration (1.33±0.33) compared to the untreated diabetic group (3.33±0.33, p=0.004) and the diabetic group treated with NAC (2.83±0.31, p=0.026). The group treated with glycine also showed significant reducement in septal infiltrates (1.33±0.49) compared to the untreated diabetic group (p=0.015) and the diabetic group treated with NAC (p=0.041).

The role of NAC in mitigating lung injury has been an area of active research, particularly in conditions such as diabetes mellitus and lung fibrosis. In our study, we explored the impact of NAC, both alone and in combination with glycine, on lung tissue inflammation and fibrosis in a diabetic rat model. Our findings were compared with a recent study, which investigated the antifibrotic effects of NAC on airway smooth muscle cells treated with Transforming Growth Factor Beta 1 (TGF-β1) [14].

The results of our study revealed that combining NAC with glycine resulted in a notable decrease in peribronchial, peribronchiolar, and septal infiltrates in diabetic rats. These findings indicate that NAC, especially when combined with glycine, can possess beneficial anti-oxidative and anti-inflammatory effects in the setting of diabetic lung damage. Compared to NAC alone, the combined therapy resulted in a more significant decrease in inflammatory infiltrates, suggesting a possible synergistic impact of NAC and glycine. Although there were some differences in volume densities of alveolar spaces and volume densities of the septal connective tissue among the various treatment groups, they were not statistically significant, as we would have expected, based on our qualitative histological analysis. One possible reason might be the small sample size that was used in this study. On the other side, it might also indicate that the main impact of NAC in this model is likely more on inflammation rather than on connective tissue accumulation in lung tissue, which was also supported by the results from the study by Raghu et al. [15].

The results of our study can be partially compared with the results from a previously mentioned study by Raghu et al. as well as the study conducted by Zhu et al. The researchers found that NAC inhibited the fibrotic effects of TGF-β1 on airway smooth muscle cells by reducing mitochondrial ROS production. This antioxidant effect of NAC led to decreased activation of pathways associated with fibrosis and inflammation, ultimately reducing cellular fibrosis and inflammation in the airway smooth muscle cells [14,15].

The results of our study can also be compared with the results of a systematic review by Tenorio et al. The review evaluated numerous studies and demonstrated that NAC has a substantial effect on reducing inflammatory markers and immune cell infiltration in various models, including those induced by compounds such as lipopolysaccharide (LPS) and mechanical ventilation. The consistency of these results underscores NAC's potential to effectively regulate inflammatory responses in lung tissue. Additionally, NAC's protective effects are further enhanced by its ability to mitigate oxidative stress, which is closely associated with tissue injury and inflammation [16].

While our study did not specifically explore the mechanisms underlying the observed effects, the anti-inflammatory outcomes are likely linked to NAC’s well-documented antioxidant properties [17]. A previously mentioned study by Tenorio et al. provides a deeper insight into these mechanisms, noting that NAC reduces oxidative stress by scavenging ROS and enhancing the synthesis of glutathione, an essential antioxidant [16]. Although direct measurements of oxidative stress markers were not included in our study, the reduction in inflammation can be indirectly attributed to a decrease in oxidative stress, given the close relationship between these two phenomena [18].

A study by Huang et al. in a mouse model of silicosis emphasizes NAC’s effectiveness in reducing oxidative markers such as malondialdehyde (MDA) and boosting antioxidant defenses like superoxide dismutase (SOD) activity and glutathione levels, suggesting that NAC’s action in reducing oxidative stress likely contributed to the mitigation of lung inflammation and lung fibrosis in the mouse model study, where the reduction of oxidative stress was crucial [19].

Our research highlighted that NAC treatment resulted in a noticeable decrease in the histopathological hallmarks of fibrosis, such as collagen deposition and the thickening of alveolar walls. While we did not measure fibrosis per se, the significant decrease in inflammatory infiltrates suggests a potential for NAC to also impact fibrotic changes, particularly since inflammation and fibrosis are often interconnected in pulmonary diseases. This suggests that NAC could be similarly effective in reducing early fibrotic changes, a hypothesis that would need further investigation in our model.

Besides studies analyzing the impact of NAC on different medical conditions, there have been a lot of studies analyzing amino acids as a treatment method for various diseases [6,9,19,20]. In contrast to our research, the study by Xiaoshi Ma and colleagues explored the effects of functional amino acids on apoptosis, inflammatory response, and pulmonary fibrosis in mice subjected to LPS to induce acute lung injury. This research demonstrated that certain amino acids could modulate key pathways involved in cellular stress responses, effectively reducing apoptosis, inflammation, and subsequent fibrotic changes in lung tissues [20]. 

Although our study presents evidence of decreased inflammatory cell infiltrations in the lungs of diabetic rats, the previously mentioned study by Ma et al. expands on this knowledge by demonstrating that functional amino acids can also suppress inflammatory cytokines and markers. These factors are directly associated with decreased fibrotic deposition in the lungs. This observation is consistent with our results, indicating various potential uses of amino acids in treating pulmonary inflammation. The same research provides insight into the regulation of apoptosis, which was not directly evaluated in our study. Functional amino acids have been shown to reduce the apoptosis rate in lung cells, a critical factor in protecting against the progression of inflammatory and fibrotic processes after acute lung injuries, such as those caused by LPS [19,20]. 

A comprehensive understanding of the therapeutic potential of amino acids, such as NAC and glycine, relies on this crucial element of cellular defense. Future research should aim to bridge the gaps between these findings, potentially exploring the combined effects of various amino acids in a single comprehensive study to harness their full therapeutic potential in pulmonary medicine.

Furthermore, glycine has emerged as a promising therapeutic agent in treating various other chronic conditions [21]. Another study highlighted glycine's role in treating nonalcoholic fatty liver disease (NAFLD) by influencing several key metabolic processes including enhanced fatty acid oxidation and reduced liver fat accumulation, as well as improved glutathione levels and strengthen antioxidant defenses, which are pivotal for maintaining cellular health in the liver [22]. Furthermore, Aguayo-Cerón et al. emphasize the efficacy of glycine in mitigating inflammatory reactions in several physiological systems including metabolic disorders and neurological diseases by its capacity to regulate cytokine synthesis [23]. Our research also confirmed glycine as potent in reducing fibrosis and inflammation and more potent than NAC in reducing septal infiltrates. However, in our study glycine was more potent when used in combination with NAC. This is in line with the results of another study by Kumar et al. that explored the combined use of NAC and glycine, also abbreviated to GlyNAC [24]. Their study demonstrated encouraging outcomes in treating physiological impairments linked to aging, including increased glutathione levels, decreased oxidative stress and inflammation, improved mitochondrial function, and enhanced overall physical function.

Although we provided valuable insights into the efficacy of glycine and the even higher efficacy of glycine combined with NAC in the management of diabetic pulmonary complications, our study had several limitations. First, the sample size was small, limiting the findings' statistical power and generalizability. We also used fixed doses of NAC and glycine without exploring a range of doses or treatment durations. Finally, the short study duration of 12 weeks may not capture the long-term effects of the treatments.

Nevertheless, more studies are needed to further explore the therapeutic potential of the combination of NAC and glycine in controlling diabetes-related complications and other medical disorders.

## Conclusions

Although NAC and glycine exhibit limited anti-inflammatory therapeutic potential when administered separately, their combined application proved to be significantly effective in alleviating pulmonary fibrosis and inflammation in diabetic rats, suggesting a possible synergistic interaction between the two agents. This indicates that although each medication may possess restricted potency individually, their combined use could improve therapeutic results. Further research is necessary to assess the efficacy and safety of these drugs, individually and in combination, in preventing or mitigating pulmonary problems linked to type 1 diabetes and other fibrotic disorders. This research is essential for using these discoveries in clinical practice, where comprehending optimum dosages, modes of action, and long-term consequences is critical for formulating effective treatment regimens.
